# Safety, pharmacokinetics and resistant variants of telaprevir alone for 12 weeks in hepatitis C virus genotype 1b infection

**DOI:** 10.1111/j.1365-2893.2011.01514.x

**Published:** 2012-02

**Authors:** I Yamada, F Suzuki, N Kamiya, K Aoki, Y Sakurai, M Kano, H Matsui, H Kumada

**Affiliations:** 1 Development Division, Mitsubishi Tanabe Pharma CorporationTokyo, Japan; 2 Department of Hepatology, Toranomon HospitalTokyo, Japan

**Keywords:** genotype 1b, pharmacokinetics, resistant variants, telaprevir monotherapy, tolerability

## Abstract

*Background:* Telaprevir in combination with peginterferon and ribavirin is a promising advancement in chronic hepatitis C treatment. However, the safety, tolerability, pharmacokinetics and antiviral profiles of telaprevir alone beyond 2 weeks have not been studied. *Methods:* In a phase 1b study in Japan, 10 treatment-naïve patients infected with hepatitis C virus genotype 1b with high viral load (>5 log_10_ IU/mL) received telaprevir 750 mg every 8 h (q8h) for 12 weeks. We examined the safety, tolerability, pharmacokinetics, hepatitis C virus (HCV) RNA levels and resistant variants of telaprevir. *Results:* Neither serious adverse events nor discontinuations of study drug owing to an adverse event occurred. The most common adverse drug reactions were rash (80%) and anaemia (70%). Telaprevir concentration reached its steady state within 2 days after the first administration without abnormal accumulation. Telaprevir alone provided potent antiviral activity: a median log_10_ decrease of 2.325 at 16 h and 5.175 on Day 14. During the treatment, HCV RNA levels at the nadir were below the limit of the quantification in seven patients and undetectable in three of 10 patients. Viral breakthrough associated with mainly Ala^156^-substituted variants occurred in eight patients, and only one patient showed end-of-treatment response. The selected variants reverted to the wild-type during the 24-week follow-up period. *Conclusion:* Telaprevir alone was well tolerated at 750 mg q8h for up to 12 weeks. The safety profile and emergence of resistant variants of genotype 1b under telaprevir monotherapy for 12 weeks will become increasingly important in evaluating an oral combination of telaprevir with other direct-acting antiviral agents.

## Introduction

Hepatitis C virus (HCV) infection often causes chronic hepatitis (CHC) that may result in life-threatening complications including cirrhosis and hepatocellular carcinoma (HCC) [1,2]. Thus, the development of medical agents or therapies that are highly effective against HCV has been eagerly sought for a long time. The current standard of care (SOC) for patients with hepatitis C, the concomitant administration of peginterferon (PEG-IFN) with ribavirin (RBV) for 48 weeks, is one such therapy, but it results in sustained virological response (SVR) in only about 45% of patients with genotype 1 HCV infection [3–5]. In addition to this low rate of SVR, another large problem of the SOC is that its practical use has been often interrupted or discontinued with several side effects including flu-like symptoms, depression, neutropenia and anaemia, and some patients are also excluded from SOC. Patients not eligible for SOC include many with comorbid conditions that often accompany HCV, including decompensated liver disease and renal failure. Thus, there is an unmet need for CHC therapies that are more effective and are better tolerated than what is presently available. Telaprevir, which is a novel peptidemimetic slow and tight-binding inhibitor of the HCV NS3-4A protease discovered using a structure-based drug design approach [6], has been intensively developed in the world as a member of a new class of direct-acting antivirals (DAAs) to improve SVR rates for genotype 1. In the first, phase 1 trial (VX04-950-101) in CHC patients, telaprevir was well tolerated and reduced HCV RNA in plasma by 2 log_10_ or greater after its consecutive administration for 14 days [7]. In a subsequent phase 1 clinical trial (VX05-950-103), all eight patients given telaprevir alone had an initial, rapid and profound antiviral response, but the four patients with genotype 1a infection experienced a viral breakthrough, whereas the other four patients with genotype 1b infection had a continuous decline in viral load [8]. Because genotype 1b infection accounts for 70% of patients and genotype 1a is rarely met with in Japan [9], viral kinetics and emergence of resistant variants from telaprevir use alone beyond 2 weeks remain to be evaluated among patients with genotype 1b infection. Besides virological reasons, a safer therapy without concomitant administration of PEG-IFN or RBV is desirable if possible, because the majority of HCV carriers are of age >55 years whose tolerability is of concern in Japan [10]. Therefore, the purpose of this trial is to examine the safety, tolerability, antiviral effects and pharmacokinetics of monotherapy with telaprevir in 10 Japanese patients with genotype 1b infection for up to 12 weeks.

## Patients and methods

### Study design and organization

This single-arm, open-label study was conducted from December 2007 to October 2008 at the Department of Hepatology in the Toranomon Hospital in Metropolitan Tokyo in full compliance with the guideline of Good Clinical Practice and the Declaration of Helsinki (ClinicalTrials.gov Identifier: NCT00591214). Before the study started, the protocol and informed consent forms were reviewed and approved by the institutional review board. Informed consent was obtained from all patients in writing after sufficient explanation was given and before they participated in the study. For 12 consecutive weeks, all 10 patients received 750 mg telaprevir q8h under feeding conditions. Telaprevir was supplied as a 250-mg tablet.

### Patients

Patients enrolled in this study were treatment-naïve, HCV-infected male or female participants with characteristics shown in Table 1, who met the following inclusion criteria: diagnosed with chronic hepatitis C; infected with HCV genotype 1b proved by phylogenetic analysis in the NS5B region; not received any prior antiviral therapy for hepatitis C; had HCV RNA level of 5 log_10_ IU/mL or more determined by the Roche COBAS TaqMan HCV test (Roche Diagnostics, Tokyo, Japan); belonged to Japanese race (Mongoloid) aged from 20 to 65 years at entry; and agreed birth control from the time of obtaining informed consent to 24 weeks after the completion of administration of the study drug. Patients were excluded from the study if they met any of the following criteria: diagnosed with decompensated liver cirrhosis and/or presence of hepatitis B surface antigen in serum; diagnosed with HCC or its history; previously treated for malignant neoplasm; diagnosed with autoimmune hepatitis, alcoholic liver disease, haemochromatosis, or chronic liver disease other than chronic hepatitis C; women who were pregnant, were breast-feeding, or who could become pregnant; had a history of alcohol addiction; and had complications of heart, kidney and lung disease.

**Table 1:** Patient characterstics, treatment duration, and viral response

	Sex	Age	BMI (kg/m^2^)	Baseline hepatitis C virus (HCV) RNA (Log_10_IU/mL)	Treatment duration (day)	HCV RNA Nadir (Log_10_IU/mL)	Viral response
1	M	31	29.1	7.10	58*	1.6	Breakthrough
2	M	64	30.7	6.70	50*	<1.2 detectable	Breakthrough
3	M	48	25.7	5.10	63*	Undetectable	Breakthrough
4	M	49	22.7	6.60	45*	3.0	Breakthrough
5	F	64	24.2	6.95	85 (completed)	1.2	Partial responder†
6	M	58	19.7	6.50	63*	<1.2 detectable	Breakthrough
7	F	63	22.8	6.40	58*	<1.2 detectable	Breakthrough
8	M	49	22.6	5.50	87 (completed)	Undetectable	Relapser
9	M	59	21.2	6.35	85 (completed)	Undetectable	Breakthrough
10	F	55	19.0	6.25	51*	<1.2 detectable	Breakthrough

Subjects whose viral level increased by 2 Log_10_IU/mL from nadir or more than 3 Log_10_IU/mL after reaching undetectable levels during treatment phase are defined to show breakthrough.

* Subjects discontinued telaprevir due to viral breakthrough.

† Subject who did not meet both criteria of breakthrough and relapse.

### Hepatitis C virus RNA measurement

Antiviral effects of telaprevir on HCV were assessed by measuring the serum HCV RNA levels using the COBAS TaqMan HCV test (Roche Diagnostics). Blood samples were collected on Day-28, before dosing (0 h) and 2.5, 4, 8, 16 and 24 h after the first dosing on Day 1 and before dosing on Days 3, 8, 14, 29, 43, 57 and 86 and at Weeks 1, 2, 4, 8, 12, 16, 20 and 24 after the end of treatment. The linear dynamic range of the assay was 1.2 to 7.8 log_10_ IU/mL. The qualitative result below the lower limit of quantification (LLOQ) was also determined as positive (1.0) and negative (0.5).

### Sequence analysis of the hepatitis C virus NS3 protease domain

Hepatitis C virus RNA was isolated from serum samples collected on Day-28, and Days 1, 3, 8, 14, 29, 43, 57 and 86 and at Weeks 1, 2, 4, 8, 12, 16, 20 and 24 after the end of treatment. The DNA fragment of 534 bp in length (181 amino acids) encompassing the NS3 protease domain was amplified by nested RT-PCR and cloned. At least 39 clones per specimen were sequenced bidirectionally. The limit of detection (LOD) for sequencing analysis was around 3 log_10_ IU/mL.

### Safety assessments

Safety and tolerability of study treatments were assessed by clinical laboratory results, vital signs, 12-lead electrocardiograms (ECGs) and occurrence of adverse events. These safety parameters were recorded at regular intervals from Day-28 through the follow-up visits.

### Determination of pharmacokinetic parameters

Blood samples were collected immediately before dosing (0 h) and 1, 2.5, 4, 6, 8, 12, 16 and 24 h after the first dosing on Days 1, 14 and 85 and before dosing on Days 3, 8, 29, 43 and 57. Plasma concentrations of telaprevir were determined using a high-performance liquid chromatographic apparatus fitted with mass spectrometry. Plasma concentrations and actual plasma-sampling times were used to calculate the area under the plasma concentration–time curve from 0 to 8 h (AUC_0–8 h_) and terminal half-life (*t*_1/2_) by the noncompartmental method using WinNonlin software version 5.2.1. The maximum plasma concentration (*C*_max_) and time to reach *C*_max_ (*t*_max_) were directly determined from the observed values on Days 1, 14 and 85.

### Statistical analysis

From the plasma concentrations of telaprevir, descriptive statistics were calculated. The number of patients with adverse events was summarized by MedDRA (version 11.1.) system organ class, preferred term, severity and relationship to study drug. All statistical analyses were performed using the validated version 9.1.3 of the SAS® System (SAS Institute Inc., Cary, NC, USA).

## Results

### Baseline characteristics

A total of 10 Japanese patients, whose background characteristics are shown in Table 1, were enrolled in this study. Their median age was 56.5 years (range, 31–64), and 7 (70.0%) and 3 (30.0%) were men and women, respectively. Baseline HCV RNA levels of each subject were similar in the range 5.10 log_10_–7.10 log_10_ IU/mL (median: 6.450).

### Safety and tolerability

There were neither serious adverse events nor discontinuations owing to an adverse event. In the present study, 75 adverse events and 66 adverse drug reactions, respectively, developed in nine of 10 patients (90.0%). An incidence of adverse events that developed in two or more patients by the preferred terms is shown in Table 2. The adverse events with the incidence of 30% or higher were rash developing in eight patients (80.0%) (if pruritic rash is included in rash, nine patients [90.0%]), anaemia in seven patients (70.0%), blood uric acid increased in five patients (50.0%), low-density lipoprotein increased in five patients (50.0%), stomach discomfort in four patients (40.0%), peripheral oedema was present in three patients (30.0%), blood triglycerides increased in three patients (30.0%), and pruritus was seen in three patients (30.0%). The moderate adverse events (one each) that developed in five patients were vertigo, peripheral oedema, nasopharyngitis, increase in blood uric acid and in low density lipoprotein, facial palsy and rash, whereas all other adverse events were mild. It is notable that although seven patients discontinued the therapy, none did so owing to adverse events.

**Table 2:** Incidence of adverse events that occurred in two or more patients

	*N* = 10			
	
	Mild	Moderate	Severe	Total
				
	*N* (%)	*N* (%)	*N* (%)	*N* (%)
Subjects with adverse events	9 (90.0)	5 (50.0)	0 (0.0)	9 (90.0)
Rash	7 (70.0)	1 (10.0)	0 (0.0)	8 (80.0)
Anaemia	7 (70.0)	0 (0.0)	0 (0.0)	7 (70.0)
Blood uric acid increase	4 (40.0)	1 (10.0)	0 (0.0)	5 (50.0)
Low-density lipoprotein increase	4 (40.0)	1 (10.0)	0 (0.0)	5 (50.0)
Stomach discomfort	4 (40.0)	0 (0.0)	0 (0.0)	4 (40.0)
Blood triglycerides increase	3 (30.0)	0 (0.0)	0 (0.0)	3 (30.0)
Pruritus	3 (30.0)	0 (0.0)	0 (0.0)	3 (30.0)
Peripheral Oedema	2 (20.0)	1 (10.0)	0 (0.0)	3 (30.0)
Malaise	2 (20.0)	0 (0.0)	0 (0.0)	2 (20.0)
Pyrexia	2 (20.0)	0 (0.0)	0 (0.0)	2 (20.0)
Nasopharyngitis	1 (10.0)	1 (10.0)	0 (0.0)	2 (20.0)

### Antiviral activity

**Fig. 1:**
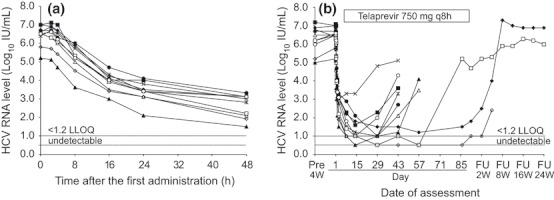
Changes in patient hepatitis C virus (HCV) RNA level. For 12 consecutive weeks, all 10 patients received 750 mg telaprevir q8h under feeding conditions. <1.2 LLOQ, below lower limit of quantification of 1.2 log_10_ IU/mL; FU, follow-up.

Telaprevir rapidly decreased serum HCV RNA level in all patients enrolled in this study. The median serum HCV RNA level changed from 6.45 log_10_ IU/mL (range: 5.1–7.1) just before administration to 4.00 log_10_ IU/mL (range: 3.0–4.7) at 16 h after administration and 1.10 log_10_ IU/mL (range: 0.5–3.3) on Day 14 (Fig. 1). Telaprevir showed potent antiviral activity: a median log_10_ decrease of 2.325 at 16 h and 5.175 on Day 14. During the administration period of 12 weeks, HCV RNA levels decreased to less than the LLOQ of 1.2 log_10_ IU/mL in seven patients, and three patients achieved HCV RNA negativity on Day 14 or Day 29. After the decrease in serum HCV RNA, breakthrough occurred in eight patients, and seven of those patients discontinued the trial during the dosing period (from Day 45 to Day 63, Table 1). In addition, one of the remaining three patients who completed the administration of the study drug achieved virus negativity by the end of administration (Day 86), but relapsed 1 week after completion of drug therapy.

### Hepatocyte injury markers

**Fig. 2:**
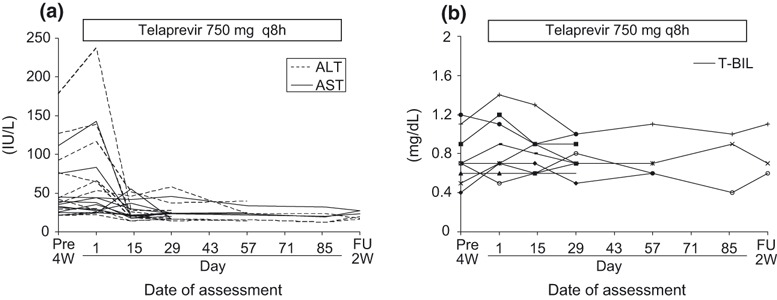
Change in alanine aminotransferase, aspartate aminotransferase (a) and total bilirubin (b) levels. FU, follow-up.

As shown in Fig. 2a, the alanine aminotransferase (ALT) and aspartate aminotransferase (AST) levels decreased during and after telaprevir treatment. Median changes from baseline (Day 1) in ALT and AST were −26.5 IU/L (range: −217–5, *N* = 10) and −8.5 IU/L (range: −118–2, *N* = 10) on Day 29, respectively. Fig. 2b shows total bilirubin levels. No clinically significant change in bilirubin was observed in all patients. These data indicate that long-term exposure to telaprevir caused neither damage nor injury in the liver.

### Sequence analysis of hepatitis C virus NS3

Amino acid substitutions in the NS3 protease domain, which were selected by telaprevir administration, were examined in 39 clones or more for each sample (Table 3). The predominant variants detected during the early time points after administration (on Days 3 and 8) were V36G, T54A and A156V. Subsequently, these variants decreased below the LOD in nine patients, and the predominant variants detected at viral breakthrough after Week 6 of administration (Day 43–86) were single-substituted variants of A156F/T/V and multiple-substituted variants of T54S+A156T and A156T+V158I; no wild-type virus was detected. In the three patients who completed the administration of telaprevir for 12 weeks, V36A, T54A and T54S+A156S/T were detectable after treatment. In the two patients followed up for 24 weeks, gradual enrichment of the wild-type viruses was observed.

**Table 3:** Means of hepatitis C virus (HCV) RNA levels and representation rates of variants in all subjects during and after telaprevir treatment

	Pre	Day 3	Day 8	Day 14	Day 29	Day 43	Day 50–60	Day 86	FU2W	FU4W	FU8W	FU12W	FU24W
													
*N*	10	10	10	10	10	10	9	3	3	3	2	2	2
Mean of HCV RNA level (log_10_ IU/mL)	6.35	2.61	1.84	1.45	1.56	2.53	3.22	2.40	2.90	4.13	6.60	6.45	6.45
HCV NS3 variants (%)													
Wild	100.0	40.0	0.2	–	0.2	0.1	–	–	–	18.3	86.4	51.8	97.8
V36A	–	–	–	–	–	–	0.3	–	–	23.8	3.4	38.5	1.1
V36G	–	10.0	0.4	–	2.4	0.8	–	–	–	–	–	–	–
T54A	–	–	9.4	9.5	4.7	0.1	–	–	–	17.9	1.1	5.0	–
A156F	–	–	–	–	–	10.0	25.5	0.8	–	–	–	–	–
A156T	–	–	–	0.5	–	7.5	16.6	31.1	16.3	–	–	1.2	–
A156V	–	–	30.0	–	1.1	15.9	2.3	–	–	–	–	–	–
T54S+A156S	–	–	–	–	–	–	–	–	–	19.2	3.4	1.3	–
T54S+A156T	–	–	–	–	–	9.6	11.4	1.5	16.3	15.0	–	–	–
A156T+V158I	–	–	–	–	–	3.3	10.1	–	–	–	–	–	–

–, not detected; FU, follow-up. Minor substitutions (maximum occupancy in a specimen was less than 10%): T54S, R155G, R155L, A156S, V36A+T54A, V36A+A156S, V36G+A156V, T54A+R155L, T54A+A156S, T54A+A156V, T54S+R155L, T54S+A156V, T54A+V132L, A156S+V132L, T54A+V163I, T54S+A156T+V158I, V36A+T54A+A156S

### Pharmacokinetics

**Fig. 3:**
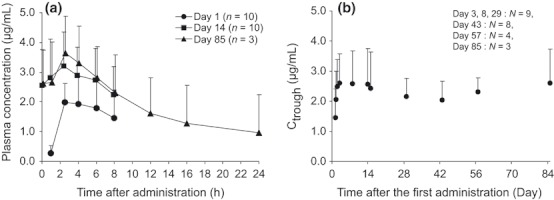
Time course of plasma concentration (a) and *C*_trough_ (b) of telaprevir. Symbols and error bars indicate mean values and SD, respectively.

The plasma concentration *vs* time curves on Days 1, 14 and 85 are shown in Fig. 3a and the *C*_trough_ on Days 1, 2, 3, 8, 14, 15, 29, 43, 57 and 85 in Fig. 3b. The pharmacokinetic parameters of telaprevir on Days 1, 14 and 85 are given in Table 4.

**Table 4:** Pharmacokinetic parameters of plasma telaprevir

	N	*C*_max_ (μg/mL)	*t*_max_ (h)*	AUC_0–8 h_ (μg·h/mL)	*C*_trough_ (μg/mL) †	*t*_1/2_ (h)
Day 1	10	2.24 ± 0.93	2.50 (2.30–7.92)	11.60 ± 4.74	1.462 ± 0.949	5.57 ± 2.67‡,§
Day 14	10	3.34 ± 1.11	2.49 (0.98–5.97)	22.31 ± 8.29	2.239 ± 0.953	9.64 ± 6.14‡,¶
Day 85	3	3.68 ± 1.29	2.72 (2.68–4.00)	23.98 ± 9.45	2.312 ± 1.265	18.35 ± 22.91**

Mean value ± SD.

* Median (minimum value to maximum value).

† *C*_trough_ at 8 h after the first administration.

‡ Calculated from measured values at 8 h after the first administration.

§ *N* = 7.

¶ *N* = 8.

** Calculated from measured values at 24 h after the first administration.

As the t_max_ were similar on Days 1, 14 and 85 with medians of 2.50, 2.49 and 2.72 h, respectively, the repeated administration under the present conditions was unlikely to cause any change in absorption. The pharmacokinetic parameters of *C*_max_, AUC_0–8 h_ and *C*_trough_ were lower on Day 1 than those on Days 14 and 85; thus, on Days 1, 14 and 85, the mean values of *C*_max_ were respectively 2.24, 3.34 and 3.68 μg/mL, the mean values of AUC_0–8 h_ were respectively 11.60, 22.31 and 23.98 μg·h/mL, and the mean values of *C*_trough_ at 8 h after the first administration were respectively 1.462, 2.239 and 2.312 μg/mL. The plasma concentration of telaprevir reached steady state on Day 2.

## Discussion

During the past decade, the combined use of PEG-IFN and RBV has provided a significant therapeutic advance for patients with CHC. Approximately 50% of patients infected with genotype 1 HCV do not, however, achieve SVR with this SOC [3–5]. On the contrary, the treatment with telaprevir-based triple regimen significantly improved SVR rates in patients with genotype 1 HCV. The PROVE 1 and 2 studies of telaprevir use with PEG-IFN and RBV in treatment-naïve patients achieved SVR rates of 61% and 69% (placebo: 41–46%) [11,12]. The Japanese study of the telaprevir-based triple regimen also showed high SVR rates [13–15]. However, the key safety concerns with the telaprevir-based triple regimen were anaemia, rash and IFN-induced systemic symptoms, all of which were most likely caused by the PEG-IFN/RBV treatment. In Japan, there are currently a large number of aged people with genotype 1b HCV and high viral loads, which is one of the most intractable HCV genotypes. As a result of advanced age, many subjects could not tolerate the adverse drug reactions in the telaprevir-based regimen, which was also observed with PEG-IFN/RBV therapies [13,15]. This observation prompted us to re-examine the safety profiles and pharmacokinetics of monotherapy with telaprevir for 12 weeks in Japanese patients.

In this study, 10 treatment-naïve patients with genotype 1b CHC and a high median viral load of 6.45 log_10_ IU/mL (range: 5.10–7.10) (Table 1) took 750 mg of telaprevir q8h for 12 weeks under feeding conditions. The plasma concentrations of telaprevir reached steady state within 2 days after the initiation of administration in the 750-mg q8h regimen as is shown by the constant *C*_trough_ from Day 2 to Day 85; hence, all the patients enrolled in this study were sufficiently exposed to telaprevir during treatment (Fig. 3b). These results demonstrate that the plasma concentrations of telaprevir were manageable even during the long-term repeated administration. There were no clinically significant events, although the incidence of some events exceeded 20.0% (Table 2). Notably, mild anaemia developed in seven patients (70%) and its occurrence was consistent with the decrease in haemoglobin values, although gradual, during the first 29 days after administration of telaprevir. The incidence of rash, which is reported to develop with a high incidence and high severity in the clinical trials of co-administration of telaprevir with PEG-IFN and RBV [11,12], was also high but its severity was mild in this study. Although exposure to telaprevir was sufficient to eliminate the virus, neither serious adverse events nor discontinuations because of adverse events occurred during the study period. The results confirmed the high tolerability of telaprevir alone after long-term administration. Although there has been no direct comparison of telaprevir monotherapy and telaprevir-based triple therapy, based on these results, the severe adverse drug reactions reported for telaprevir-based triple therapy including anaemia and rash were likely to be ascribed to the synergistic and/or additive effects of the three drugs, i.e., telaprevir, PEG-IFN, and RBV. The safety information under telaprevir monotherapy described here is very important to understand the aspects of adverse drug reactions, especially anaemia and rash, in telaprevir-based triple therapy. In addition, compared to baseline, the ALT and AST levels were significantly lower during the treatment in all patients, indicating that telaprevir was unlikely to cause direct liver damage or injury even after long-term use.

Although there is a report on HCV RNA mutation after monotherapy with a protease inhibitor for 14 days [16], no information about the selective pressure of such protease inhibitors administered alone for a longer period is available at present. During the treatment period in this study, HCV RNA levels were below the LLOQ in seven patients and undetectable in three patients. Importantly, one patient showed an end-of-treatment response. Viral breakthrough resulting from the selection of Ala^156^-substituted variants with high-level resistance to telaprevir [16] occurred in eight patients. It has been reported that high-level resistance was absent, low-level resistance was minimized, and the majority of the viral population reverted to the wild-type by 3–7 months after telaprevir dosing for 14 days [16]. In the two patients who were studied up to the last visit, enrichment of the wild-type viruses was observed at Week 24 of the follow-up period. It is thus clear that the variants that appeared during prolonged administration of telaprevir for 12 weeks could be replaced by or could revert to the wild-type viruses. This study also provides new knowledge about a selective pathway of the NS3 protease domain of HCV genotype 1b during long-term telaprevir administration (Table 3). It is notable that the wild-type viruses were eliminated promptly by Day 3 of telaprevir monotherapy in all cases, but variants with amino acid substitutions such as V36G, A156V and T54A still remained on Days 3 and 8. From Day 50 to Day 99, A156T was the predominant variant after viral breakthrough. On Day 43, several substitutions that are rarely reported were found: a single substitution of A156F and multiple substitutions of T54S+A156T and A156T+V158I. In the clonal sequencing analysis in this trial, the observed T54S and V158I substitutions were mostly associated with the A156S/T substitution, and enrichment of multiple-substituted variants was observed under prolonged telaprevir treatment (Fig. s1). A phenotypic enzyme assay suggested that the solo T54S substitution did not change the inhibitory concentration of telaprevir (data not shown). It has also been reported that the T54S and V158I substitutions were also positively selected in the clinical trials of boceprevir, but the solo V158I substitution did not confer telaprevir resistance [17]. Therefore, these two substitutions may be a secondary resistance-associated variant of genotype 1b. Moreover, we could speculate that these variants are susceptible to PEG-IFN and RBV, because the viral variants emerging after the longer selective pressure with telaprevir monotherapy were decreased rapidly by switching the treatment with telaprevir to that with PEG-IFN and RBV [18]. Although it was reported that one patient with low viral load achieved SVR in the treatment regimen in which 750 mg telaprevir was administered q8h for 24 weeks [19], no patients with high viral load achieved SVR in this study. As discussed earlier, PEG-IFN and RBV-free therapy is an unmet and strong medical need in Japan. Therefore, an oral cocktail therapy for HCV genotype 1b infection using telaprevir and different types of DAAs, for example HCV NS5A or NS5B polymerase inhibitors, would be warranted to improve efficacy and reduce adverse drug reactions of the telaprevir, PEG-IFN and RBV triple therapy.

In conclusion, the results of this study indicate that telaprevir is well tolerated at 750 mg q8h for 12 weeks in Japanese patients with HCV genotype 1b infection. The data obtained in this study on telaprevir monotherapy demonstrate that the severe side effects, rash and anaemia observed in the telaprevir-based triple regimen were likely to be attributable to the additive and/or synergistic effect of telaprevir, PEG-IFN and RBV, and this consideration has encouraged us to evaluate telaprevir in a combination therapy with a different class of DAAs in future.

## Abbreviations

ALT: alanine aminotransferase
AST: aspartate aminotransferase
CHC: chronic hepatitis C
DAA: direct acting antiviral
HCC: hepatocellular carcinoma
HCV: hepatitis C virus
LLOQ: lower limit of quantification
LOD: limit of detection
PEG-IFN: peginterferon
q8h: every 8 h
RBV: ribavirin
SOC: standard of care
SVR: sustained virological response

## SUPPORTING INFORMATION

Figure S1: Pie chart of variant occupation at each time point in the typical two cases. Proposed secondary resistant associated substitutions are underlined.
